# Organizational interventions improving access to community-based primary health care for vulnerable populations: a scoping review

**DOI:** 10.1186/s12939-016-0459-9

**Published:** 2016-10-10

**Authors:** Vladimir Khanassov, Pierre Pluye, Sarah Descoteaux, Jeannie L. Haggerty, Grant Russell, Jane Gunn, Jean-Frederic Levesque

**Affiliations:** 1 Department of Family Medicine, McGill University, 5858 Côte-des-neiges, 3rd Floor, Suite 300, Montreal, QC H3S 1Z1 Canada; 2 St. Mary’s Hospital Research Centre, 3830 Lacombe Ave, Montréal, QC H3T1M5 Canada; 3 Department of Family Medicine, McGill University, St. Mary’s Hospital Research Centre, 3830 Lacombe Ave, Montréal, QC H3T1M5 Canada; 4 Southern Academic Primary Care Research Unit, Department of General Practice, School of Primary Health Care, Monash University, Building 1, 270 Ferntree Gully Rd, Notting Hill, VIC 3168 Australia; 5 University of Melbourne, 200 Berkeley Street, Melbourne, VIC 3053 Australia; 6 Centre for Primary Health Care and Equity, University of New South Wales, Bureau of Health Information, 67 Albert Avenue, Chatswood, Sydney, NSW 2067 Australia

**Keywords:** Delivery of Health Care, Accessibility to Health Services, Vulnerable populations, Underserved populations, Organizational Interventions, Improve Access, Canada, Australia

## Abstract

Access to community-based primary health care (hereafter, ‘primary care’) is a priority in many countries. Health care systems have emphasized policies that help the community ‘get the right service in the right place at the right time’. However, little is known about organizational interventions in primary care that are aimed to improve access for populations in situations of vulnerability (e.g., socioeconomically disadvantaged) and how successful they are. The purpose of this scoping review was to map the existing evidence on organizational interventions that improve access to primary care services for vulnerable populations. Scoping review followed an iterative process. Eligibility criteria: organizational interventions in Organisation for Economic Cooperation and Development (OECD) countries; aiming to improve access to primary care for vulnerable populations; all study designs; published from 2000 in English or French; reporting at least one outcome (avoidable hospitalization, emergency department admission, or unmet health care needs). Sources: Main bibliographic databases (Medline, Embase, CINAHL) and team members’ personal files. Study selection: One researcher selected relevant abstracts and full text papers. Theory-driven synthesis: The researcher classified included studies using (i) the ‘Patient Centered Access to Healthcare’ conceptual framework (dimensions and outcomes of access to primary care), and (ii) the classification of interventions of the Cochrane Effective Practice and Organization of Care. Using pattern analysis, interventions were mapped in accordance with the presence/absence of ‘dimension-outcome’ patterns. Out of 8,694 records (title/abstract), 39 studies with varying designs were included. The analysis revealed the following pattern. Results of 10 studies on interventions classified as ‘Formal integration of services’ suggested that these interventions were associated with three dimensions of access (approachability, availability and affordability) and reduction of hospitalizations (four/four studies), emergency department admissions (six/six studies), and unmet healthcare needs (five/six studies). These 10 studies included seven non-randomized studies, one randomized controlled trial, one quantitative descriptive study, and one mixed methods study. Our results suggest the limited breadth of research in this area, and that it will be feasible to conduct a full systematic review of studies on the effectiveness of the formal integration of services to improve access to primary care services for vulnerable populations.

## Introduction

Health systems are struggling to provide equitable access to community-based primary health care (hereafter, ‘primary care’) services [1, 2]. The access to primary care services is worse for populations in situations of vulnerability (hereafter, ‘vulnerable populations’) such as poor, immigrant, and aboriginal citizens in Canada and Australia [3–6]. Emerging evidence from Canada and Australia shows that various reforms are aiming at improving access to care, but may not be well adapted to vulnerable populations [7, 8].

These access problems can pertain to the way care is offered as well as to the actual ability of people to seek, reach and engage with the care [9]. Organizational interventions might target both adjustments to the way care is delivered as well as targeting the development of people’s capacity to obtain care [9]. While access-related problems for vulnerable populations have been documented, few reviews have looked at the evidence regarding how successful programs have been at addressing access issues for these populations [10].

Knowledge remains scant about the actual scope of interventions that go beyond the establishment of specific programs aimed at improving the usual way primary care is delivered for vulnerable populations. Therefore, the purpose of this scoping review was to describe the nature and breadth of published research studies in peer reviewed academic journals on organizational interventions improving access to primary care services for vulnerable populations, and reducing consequences of poor access in these populations.

## Review

### Methods

As part of the Australian-Canadian IMPACT program (Improving Models Promoting Access-to-Care Transformation), a scoping review was chosen to (i) map relevant studies regardless of the design, theoretical rationale, and discipline; and (ii) identify a candidate research focus for a subsequent systematic review [11, 12]. Scoping reviews are used to identify knowledge gaps, set research agendas, and identify implications for decision-making. Specifically, scoping reviews are aimed to explore the breadth of available evidence in a research domain (main available research studies), and map the concepts underpinning this domain, which can lead to plan and conduct a systematic literature review if enough evidence to answer a specific question [13, 14]. Scoping reviews typically include five iterative stages: definition of the research question, identification of the relevant studies, selection of the studies, charting of the data, collating, summarizing and reporting the results. We included studies with all types of design (comprehensive approach), followed an iterative process (e.g., adjustment of the search strategy), and involved experts (the last four co-authors) throughout.

### Definition of the research questions

The specific research questions of the scoping review were as follows. What are the types of organizational interventions in primary care aiming at improving access for vulnerable populations? What are the documented impacts in terms of avoidable hospital admissions, emergency department presentations and unmet needs for care?

### Identification of the relevant studies

This stage involved searching the following bibliographic databases: MEDLINE, Embase and CINAHL. The search strategy was designed by all co-authors, then validated and performed by a specialized librarian (example is presented in Appendix 1). The search was expanded using references in the selected studies and pertinent existing literature reviews (citation tracking). Given the scoping nature of our work, the grey literature was not searched.

Eligibility criteria were as follows: quantitative, or qualitative, or mixed methods study conducted in countries in the Organization for Economic Cooperation and Development (OECD); published in English or French between January 2000 and March 2014 (2000 was chosen as it corresponds to a shift, worldwide, towards community-based primary health care); about (i) vulnerable populations, i.e., socioeconomically disadvantaged (e.g., uninsured), racial and ethnic minorities (e.g., indigenous people), people with one or more chronic health condition (including mental illness), (ii) access-related interventions in primary care organizations, i.e., primary care setting, medical home, community health center (e.g., community mental health service), primary care services in other settings (e.g., school-based health care program), specialized care integrated in primary care settings (e.g., psychiatric team in a medical home), and (iii) evaluated impact on reduction of at least one of the following consequences of poor access: hospitalization, or emergency department admission, or unmet health care needs [9].

### Selection of the studies

This stage consisted of an iterative process in which (contrary to a systematic review process) we searched the literature, refined our search strategy based on the new findings in the identified articles (e.g., if a new organizational intervention type has been identified we included it in searched words of database - MeSH to retrieve more studies), asked experts (JH, GR, GJ, J-FL) to share their personal files, and tracked citations in selected references and literature reviews. Using the eligibility criteria, relevant publications were selected by one researcher with extensive experience in systematic reviews (VK) [15–17] and in case of doubt discussed with another researcher (PP). The selection of records (title/abstract) was very sensitive, and the selection of full-text papers was specific. It was easy to exclude bibliographic records that were obviously not relevant. In case of doubt regarding a record, the corresponding full-text paper was automatically screened. Excluded full-text papers were obviously not relevant.

### Charting the data

The following data were extracted from each included study: (i) author, year of publication, study country; (ii) study design (e.g., randomized controlled trial); (iii) study population (e.g., sample size); (iv) vulnerability context (e.g., elderly patients); (v) main characteristics of the intervention; (vi) other elements (e.g., cost); (vi) outcomes (hospitalization, emergency department admission and unmet health care needs).

### Collating, summarizing, and reporting the results

We used a three-step qualitative synthesis: (Step 1) a classification of organizational interventions, (Step 2) a classification of access dimensions and outcomes of intervention, and (Step 3) a ‘dimension/outcome’ pattern analysis. Specifically, we conducted a theory-driven qualitative content analysis to classify interventions, dimensions and outcomes [18]. For each included study, we extracted key sentences eliciting the type of intervention, dimension and outcome (derived from previous classification and conceptual framework).

### Step 1. Classification of organizational interventions

Interventions assessed in the included studies were categorized using the following financial and organizational types of intervention derived from the checklist of the Cochrane Effective Practice and Organization of Care Review Group (EPOC) [19]. The EPOC provides different categories (e.g., financial intervention) and subcategories of intervention. Based on the description of interventions in the included studies, the first author (VK) assigned them to the EPOC categories and sub-categories. Typically, this classification was straightforward. In case of doubt, the study was discussed with the second author (PP) and the final classification was based on consensus.

1. Continuity of care via case management: Coordination of assessment, treatment and arrangement for referrals.
2. Formal integration of services: Bringing together services across sectors or teams (all services at one time).
3. Clinical multidisciplinary team: Creation of a team with professionals from multiple disciplines (or new team members).
4. Continuity of care via arrangement for follow-up.
5. Revision of professional role: Shifting of roles among healthcare professionals, or expansion of role to include new tasks.
6. Institution incentive: Financial reward to the organization or providers for doing specific action.
7. Capitation: Set amount per patient.

### Step 2. Classification of access dimensions and outcomes

**Fig. 1:**
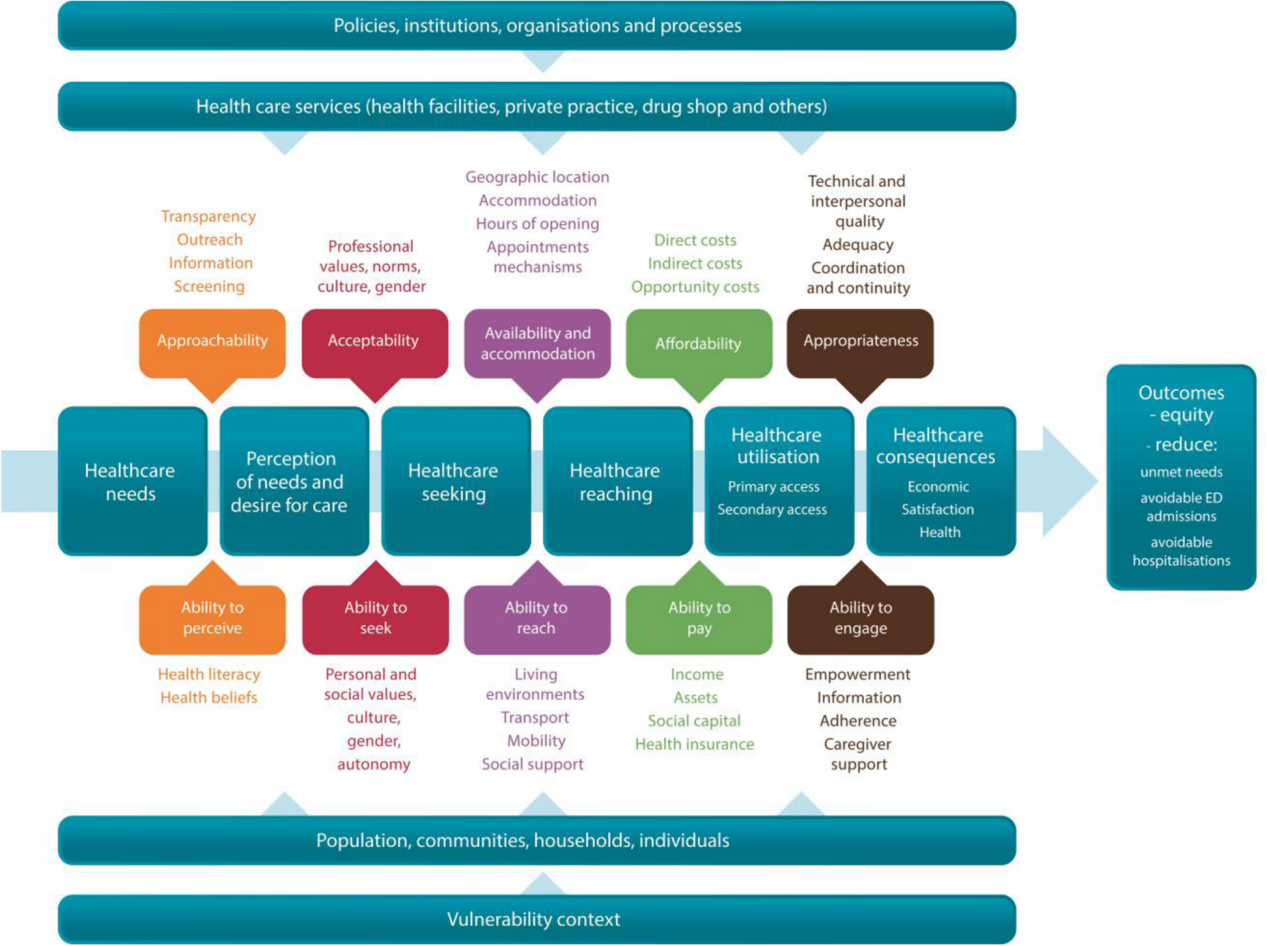
The ‘Patient Centered Access to Healthcare’ conceptual framework. Note: Conceptual framework adapted by the IMPACT program led by the last four co-authors (www.impactresearchprogram.com) from Levesque et al. [9]

Interventions assessed in the included studies were categorized using the ‘Patient Centered Access to Healthcare’ conceptual framework in terms of outcomes and dimensions [9]. First, the key outcomes were threefold: reduction of avoidable hospitalization, emergency department admission, and unmet health care needs (Fig. 1). These outcomes were chosen because they are commonly proposed in the literature and institutionalized in research funding and governmental agencies [20–23]. For example, numerous studies demonstrated that increasing access to primary care services is associated to an improvement of these three outcomes. As another example, these outcomes were chosen by the Canadian Institutes of Health Research as national priorities for primary care studies and research teams [24]. Second, the key dimensions of access were as follows.

1. Approachability: Existence of reachable services.
2. Availability: Getting services in time.
3. Affordability: Financial capacity necessary to use services.
4. Acceptability: Cultural and social acceptance of services.
5. Appropriateness: Fit between services needed and obtained.

### Step 3. ‘Dimension/outcome’ pattern analysis

Patterns were suggested when groups of studies on similar interventions were associated with similar access dimensions and similar outcomes (positive versus no effect). Within groups, each study had the same weight regardless of their design. We looked for ‘dimensions of access to primary care services and outcomes’ patterns (dimension-outcome patterns). We grouped studies that shared a given type of outcome (e.g., emergency department admission) and we searched for their shared conditions (presence/absence of each access dimension). Outcomes were categorized and coded as “positive” (reduction of avoidable hospitalization, emergency department admission, and unmet health care needs) or “no effect” (no reduction). For each group, a pattern is suggested when all studies (or almost all) had similar outcomes and access dimensions (vote counting). This pattern analysis was conducted without and with consideration of the vulnerable population type (e.g., elderly patients versus uninsured persons).

## Results

### Search results

**Fig. 2:**
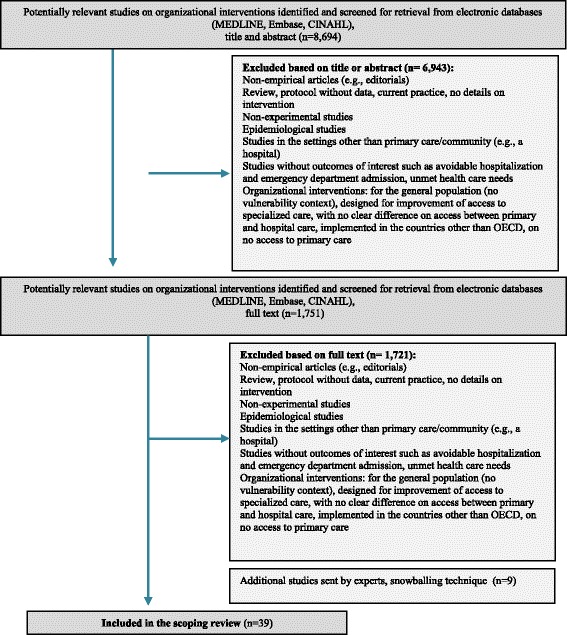
Flow Chart

The search results are outlined in a flow chart (Fig. 2). Out of 8,694 records, 6,943 were not eligible based on the title and/or the abstract, and 1,721 were excluded based on the full-text publications. An additional nine eligible studies were identified through citation tracking and personal files of researchers, leading to include 39 studies in total (Fig. 2).

### Description of included studies

Twenty-six studies were conducted in the USA [25–51], six in Canada [52–57], three in the UK [58–60], two in New Zealand [34, 61], one in Australia [62] and one in Italy [63] (Table 1). Thirty-six were quantitative studies including 11 randomized controlled trials [35–43, 49, 51, 55, 62], 22 non-randomized studies [25, 27–34, 44–48, 51, 53, 54, 58–60, 63, 64], three quantitative descriptive studies [26, 52, 61], and three mixed methods studies (Table 1) [34, 50, 56]. Twenty-five studies (64.1 %) concerned patients with chronic conditions, and 14 examined (35.9 %) socioeconomically disadvantaged populations (Table 2).

**Table 1:** Description of included studies

Author/year/ country	Study design	Population (participants and setting)	Vulnerability context	Main characteristics of the intervention	Other elements
Revision of professional roles					
Gray, 2013/ New Zealand [61]	Quantitative descriptive	Sample size: 400 Age: not reported. Patients: Children with rheumatic fever caused by the Group A Streptococcal infections. Setting: primary school	Children of primary school (1-8 years old), ethnic composition (Pacific and Maori).	*Nurse-led school-based clinics:* - social worker (ethnicity of whanau) was trained in recognition of skin infection and swabbing of sore throats; - identification of students with symptoms of a sore throat by the social worker under the supervision of the public health nurse; - medical treatment by a public health nurse (antibiotics and ointment) guided by the evidence-based guidelines; - referral of students with skin infection by the social worker to the public health nurse for the full assessment; - education of the parents on the importance and adherence to the medical treatment; - regular phone follow-up by the public health nurse; - assessment and treatment of household members at home.	Annual cost: $510 per student ($10 for consumables, $80 for diagnostic services, $420 for staffing costs).
Clinical multidisciplinary teams					
McDermott, 2001; 2004/ Australia [62]	RCT	Sample size: 21 primary healthcare centers (921 people) Age: 53.3 ± 13.6 vs 52.4 ± 14 Patients: Patients with diabetes (type 2) Setting: primary care	People with diabetes from remote indigenous communities	- implementation by the local indigenous health workers supported by a specialist outreach service in the 21 primary healthcare centers of the Torres Strait District: (i) registers of patients with diabetes; (ii) recall and reminder systems; (iii) basic diabetes care plans; - training of the local indigenous health workers in clinical diabetes care; - two-monthly newsletters.	
Doey, 2008/ Canada [54]	NRS	Sample size: 380 (survey), 805 (charts) Age: 40.7 ± 15.2 Sex (female): 51 % Patients: Patients with mental diseases Setting: community mental health clinic	Patients with mental diseases such as depression, bipolar disorder, schizophrenia, psychosis, personality disorder	*Collaborative care:* - nurse practitioner was hired to provide primary care services in collaboration with the existing team of mental healthcare professionals (including nurses, social workers, a psychiatrist, a psychologist) in a community mental health clinic; - nurse practitioner’s responsibilities were assessment and treatment of non-psychiatric acute and chronic diseases, physical examination, counseling on diet, exercise, substance abuse, - the hospitalist (PCP) from the hospital treated patients outside the nurse’s scope of practice (5 afternoons per week); - availability of the physician by phone and e-mail between visits.	External funding was allocated to hire nurse practitioners.
Crustolo, 2005/ Canada [52]	Quantitative descriptive	Sample size: 4,280 referrals annually Age: 45 % were 45-64 years old Patients: Patients with nutrition-related health conditions. Setting: primary care	Patients with dyslipidemia, type 2 diabetes, obesity.	*Shared care model of collaboration of PCP and dietitian:* - primary care practice received 10 h of nutrition services per month (half a day each week); - registered dietitian provided assessment of patients and consultation of PCP on nutrition-related problems; - patients were referred by the PCP (within 2 weeks after referral).	The Provincial Ministry of Health funded the intervention program in primary care practices.
McCuloch, 2000/USA [45]	NRS	Sample size: 15,000 (approximately) Age: not reported. Patients: Patients with diabetes Setting: Managed care (200 PCPs practicing in 25 clinics)	Patients with diabetes	*Group Health Cooperative program:* - development of electronic registry of patients with diabetes updated daily; - joint examination of patients by PCP, diabetologist, and diabetes nurse specialist (at least one visit); - application of evidence-based diabetes guidelines (retinal screening, microalbuminuria, and glycemic management; - use of patient-friendly notebook for self-management.	Decrease in diabetic per member per month costs of $62.
Michelen, 2006/USA [44]	NRS	Sample size: 1,250 (539 vs 711) Age (1-5 years): 27.1 % Ethnicity: 92.1 % of Hispanic Patients: Uninsured immigrants Setting: primary care, community health services	Uninsured immigrants with frequent use of the ED for preventable crisis.	*The Northern Manhattan Community Voices partners program:* - recruitment of a native Spanish speaker Health Priority Specialist experienced and knowledgeable of the target community and medical services; - recruitment of linguistically similar to the target population Community Health Workers; - Community Health Workers centered on direct patient and community outreach and assessment; - Community Health Workers was physically located within their community. - Health Priority Specialist were located in community medical centers; - identification of frequent users of ED and assistance to find appropriate primary care services.	
Driscoll, 2013/ USA [50]	Mixed methods study (sequential explanatory design)	Sample size: 3,213 (390 vs 2,823) Age: not reported Participants: Alaska Native/Indian population, adults with asthma Setting: primary care	The Alaska Native and American Indian population, patients with asthma	*Patient-centered medical home:* - matching of the patient to the team of medical home (self-selection or assignment); - open scheduling of the appointment; - expanded office hours; - increased ability of electronic communication between patients and healthcare professionals; - delivery of care by the multidisciplinary team: PCP, physician assistant, nurse, certified medical assistant, behavioral health consultants, nutritionists; - delegation of more authority by the physicians to non-physician members (behavioral health consultants).	
Formal integration of services					
Day, 2006/UK [65]	NRS	Sample size: 289 (126 vs 163) Age: 0-18 Diseases: Children with mental health conditions. Setting: primary care	Children 0 to 18 years old with mental health conditions	*Adolescent mental health outreach clinics:* - staffed with three clinical child psychologists, one child and family therapist; - assessment and treatment of broad range of mental health problems; - referral of patients with more complex conditions to the specialist clinics; - referral to the outreach clinics were accepted from any sources (majority from PCPs).	
Garg, 2012/USA [26]	Quantitative descriptive	Sample size: 1059 families Age: not reported. Participants: Low-income people Setting: Medical home	Low-income people	*Health lead model:* - completing a brief screening survey for social issues (e.g., food, housing) by parents at well-child care visit; - referral to the intervention team located in the pediatric clinic; - volunteer undergraduate students assist with connecting families to community-based resources through in-person meetings and telephone follow-up; - follow-up by the students; - update of referring physicians (e.g., pediatric primary care provider, nurse practitioner) on health outcomes.	
Lamothe, 2006/ Canada [56]	Mixed methods study (convergent parallel design)	Sample size: 82 Age: 75 and older Participants: Elderly patients with severe chronic conditions Setting: primary care and community (home of patients)	Elderly patients with severe chronic conditions: cardiac insufficiency, chronic obstructive pulmonary diseases, hypertension, unstable diabetes	*Telehomecare* to create a network of services between hospital and primary care providers. - equipment installed at patients’ home (a scale, thermometer, sphyngmomanometer, oxymeter, and pulse; if needed glucometer, spirometer, electrocardiograph, and a system for the measure of blood clotting); - sending of measures on a daily basis to the primary care setting; - nurse of primary care responsible for monitoring and responding to alerts from patients; - telephone and home follow-up by the nurse if needed.	
Tourigny, 2004; Hebert, 2010/ Canada [53, 64]	NRS	Sample size: 920 (501 vs 419) Age: 83 Sex (female): 67 % Participants: Elderly people Setting: primary care	Elderly people at risk of functional decline	*Program of Research to Integrate Services for the Maintenance of Autonomy (PRISMA):* - coordination between decision makers and managers at the regional and local levels; - the “single entry point” (mechanism of accessing the services such as home care, rehabilitation services, hospital services, voluntary agencies, social economy agencies); it can be access by the telephone or written referral; - 24/7 access to the general population through Health Info Line; - use of “the single assessment instrument” for evaluating needs coupled with case-mix management system; - development of the individualized service plan in collaboration of PCPs with multidisciplinary team; - computerized clinical chart to facilitate communication between organizations and healthcare professionals.	
Levkoff, 2004; Chen, 2006/USA [41, 42]	RCT	Sample size: 2,022 (999 vs 1,023) Age: 65 and older Patients: Patient with mental health conditions Setting: primary care	Patients with mental health conditions such as depression, anxiety, at risk drinking	*Integrated care model:* - co-location of mental health and substance abuse services in primary care facility; - mental health and substance abuse services include assessment, care planning, counseling, psychotherapy, pharmacological treatment); - PCPs required to be closely involved in the patient’s care.	
Brown, 2005/ USA [29]	NRS	Sample size: 17 Age: 41 Sex (female): 65 % Patients: Patients with mental health problems Setting: primary care	Patients with psychiatric health conditions (e.g., depression, panic disorder) and with high level of medical admission, ED visits, frequent outpatient visits, and frequent telephone calls.	*Primary intensive care:* Integration of mental health services in primary care facility: - location of an internist, psychiatrist-internist, nurse practitioner, and social worker in primary care; - initial assessment (2–3 sessions) lasted longer than usual time; - multidisciplinary assessment and follow-up; - frequent visits to the clinic (weekly initially); - 24/7 availability of a team member on call via pager. - development of care plan in collaboration with PCP.	Post-intervention total hospital cost was lower (*p* = NS).
MacKinney, 2013/USA [33]	NRS	Sample size: 278 (278 vs 278) Age: not reported. Patients: Uninsured population Setting: primary care	Uninsured patients (18 years old and older) with income less than 200 % of the Federal Poverty Level	*Project Access Program (Milwaukee):* - identification of uninsured individuals via an administrative system by the county social worker; - identification of healthcare providers willing to provide free services via online, radio, newspaper public advertising; - connections of the person in need of primary care services with a provider; - delivery of full-spectrum basic laboratory and non-invasive radiology services; - no pharmacy component.	
Bradley, 2012/USA [34]	NRS	Sample size: 26,000 Age (mean): 34.2 Sex (female): 63 % Patients: Uninsured population Setting: primary care	Uninsured patients with income less than 200 % of the Federal Poverty Level	*Community-based coordinated care program:* - identification of uninsured patients in ED, outpatient or inpatient settings; - assistance with financial eligibility forms; - assignment of the primary care provider willing to provide primary care services to this category of patients; - remuneration of primary care providers: monthly management fee and fee-for-services	Over 3 years, inpatient costs per year fell by 50 % (*p* < 0.01)
Kaufman, 2000/USA [31]	NRS	Sample size: 23,143 (10,029 vs 13,114) Age (19–49): 69.5 % Sex (female): 68.6 % Patients: Uninsured patients Setting: primary care	Uninsured patients below 235 % of the Federal Poverty Level not eligible for Medicaid	*Managed care:* - relocation of county funds to primary care sites from hospitals; - assignment of eligible patients to preferred PCPs; - each patient received a care plan identification card listing his/her PCP; - monthly premium ranged from $0 to $10 for primary care visits depending upon income level; - the benefit package also includes reduced out-of-pocket cost of medications, access to 24/7 telephone triage system; behavioral health service is not covered. - increase of staff (12 new PCPs and 5 new family nurse practitioners); - extension of clinic hours; - relocation of case managers and social workers from inpatient to primary care clinics; - relocation of alcohol and substance abuse counselors to primary care clinics.	The primary care clinics received: - capitation of $4 per plan member per month as compensation; - Medicaid professional primary care services capitation rate; - reduced fee-for-service rate for specialists. Savings of $148 per member per year on the cost of outpatient and inpatient care.
Roby, 2010/USA [46]	NRS	Sample size: 2,708 (20,663 vs 34,079) Age (55 and older): 67 % Sex (female): 69 % Patients: Low-income uninsured population Setting: primary care	Uninsured patients (21–64 years old) with income less than 200 % of the Federal Poverty Level	*Medical services initiative program (a safety-net-based system):* - eligible patients are identified at the time they seek for health services; - patient is assigned to a medical home within which they choose or are assigned to the primary care provider; - patients were eligible for at least one visit to medical home within 12 months; - patients with diabetes, congestive heart failure, hypertension, asthma are required to see a doctor at least twice within 12 months; - multidisciplinary team consists of PCP, nurses, nurse practitioners, case managers/social workers; - information system connected emergency rooms and community clinics to get a history of disease by the physicians of ED; - this electronic system allowed to refer patients to their PCPs in case of nonemergent conditions; - emergency phone line staffed with registered nurses is available 24/7; - reimbursement: $15 to ED physicians for entering clinical information in the electronic system and $100 to community clinics for acceptance of referral from emergency.	PCPs are reimbursed on a fee-for-service rate based on 70 % of the Medicare fee schedule. Private providers received incentives to join the network and pay-for-performance payments for primary and preventive services.
Continuity of care via case management					
Beland, 2006/Canada [55]	RCT	Sample size: 1230 (606 vs 624) Age: 82 vs 82 Sex (female): 71 % vs 72 % Participants: Disabled elderly patients Setting: primary care	Elderly patients with chronic diseases and functional disabilities	*System of Integrated Care for Older Persons (SIPA):* Two public community organizations responsible for home care (Centre Local de Services Communautaires) conducted: - comprehensive geriatric assessment; - assessment of patients’ needs; - development of care plan in collaboration with PCP; - mobilization and delivery of community services; - availability of 24-h on-call services; - patients were followed between hospital and community.	- compensation of PCPs for their time communicating with the research team ($400 per patient annually); - 44 % higher community costs; - 22 % lower total institutional costs; - overall the intervention was neutral; - no difference in out-of-pocket costs.
Glendenning-Napoli, 2012/ USA [30]	NRS	Sample size: 83 Age (50–65): 76 % Sex (female): 60.2 % Patients: Uninsured patients Setting: primary care	Uninsured patients with one or more chronic diseases (diabetes mellitus, hypertension, congestive heart failure, coronary artery disease) with frequent admissions to the ED and hospital	*Intensive case management program:* - identification of patients with frequent use of ED and frequent hospitalizations; - in-home assessment of patient’s needs by a registered nurse (identification of barriers to accessing health care, health literacy level); - accompany of patients to PCP to engage patients in their care; - development of preventive care plan; - in collaboration with social worker identification of patient’s need for social programs; - telephone follow-up and home visits to reinforce the intervention; - in-home education sessions on available pharmacy assistance programs.	- reduction in cost for acute outpatient visits (*p* < 0.009) and inpatient hospitalizations (*p* < 0.002); - increase in cost for primary care visits (*p* < 0.02).
Leff, 2009; Boult, 2011/USA [35, 59]	RCT	Sample size: 835 (433 vs 402) Age: not reported. Patients: Older patients Setting: Managed care	Older patients (65 years and older) at high risk of using health services	*Practice-based team intervention:* - in-home comprehensive assessment of needs by a nurse (caseload 50 to 60 patients); - development of the care plan; - facilitation of the access to community resources; - monthly follow-up; - coordination of all patient care providers; - facilitation of transition between care practices; - education and support of caregivers.	Net savings (2/3 due to reductions in hospital utilization).
Shah, 2011/USA [47]	NRS	Sample size: 258 (98 vs 160) Age: 46.4 ± 9.6 vs 46 ± 10.7 Sex (female): 40.8 % vs 53.1 % Patients: Uninsured population Setting: Managed care	Uninsured Medicaid population, frequent users of ED (4 or more ED admissions, 3 or more admissions, 2 or more admissions and one ED visit within 1 year)	*Managed Care program:* - identification of uninsured frequent users of emergency room; - assignment of a personal care manager who assists with access to social and medical resources; - personal care manager helps schedule an appointment with a PCP; - personal care manager helps bridge barriers between patients and health care system; - monthly meeting of case manager with patients (at home, resource centers, at appointment); - individually developed care plan; - daily work of case manager with a patient in case of hospital admission.	Decrease of ED (*p* < 0.0001) and inpatient admission costs (*p* < 0.001)
Wang, 2012/USA [43]	RCT	Sample size: 200 (98 vs 102) Age: 42.9 ± 9.7 vs 43.6 ± 8.3 Sex (female): 8.2 % vs 3.5 % Patients: Individuals released from prison Setting: primary care	Formerly incarcerated people	*Primary care-based, complex care management:* - primary care services provided by a provider with experience working with this population and a community health worker with a personal history of incarceration ; Community health worker provides: - case management support, referrals to community-based housing, education, and employment support; - medical and social service navigation (accompanying patients to pharmacies, social services, medical and behavioral health appointments; - chronic disease self-management support (home visit for health education and medication adherence support).	The program utilized the existing resources in the community health center. The additional costs included the salary of community health worker and time of supervision.
Wohl, 2011/USA [37]	RCT	Sample size: 89 (43 vs 46) Age: not reported. Sex (female): 23.3 % vs 30.4 % Patients: Individuals with HIV released from prison Setting: community settings	Formerly incarcerated HIV patients	*Bridge case management:* - training of case managers prior to start working with incarcerated patients (focus on the identification of the talents, resources, goals in non-judgmental environment); - case managers were well aware of the services available in their home and neighboring counties; - regular meeting with incarcerated people prior to and after release to identify medical and non-medical needs; - development of care plan including housing, employment, medical care, substance abuse counseling; - transition to community case management and local services after 6 months of follow-up; - caseload of 15 clients per patient	
Dorr, 2008/USA [49]	RCT	Sample size: 3,432 (1,144 vs 2,288) Age (mean): 76.2 ± 7.2 vs 76.2 ± 7.1 Sex (female): 64.6 % vs 64.6 % Patients: Elderly patients with chronic diseases Setting: primary care	Elderly patients with chronic diseases: diabetes, depression, hypertension, congestive heart failure	*Care Management Plus:* - training of care managers (nurses) on care for seniors, caregivers, chronic disease assessment, care standards; - integration of the information technology tools (structured protocols, guidelines, tracking database) and electronic health record system in primary care facilities; - placement of care managers in primary care facilities; - referral of patients with chronic care needs by PCPs to care managers for assessment and enrolment in care management services.	
Sylvia, 2008/USA [39]	NRS	Sample size: 127 (62 vs 65) Age (mean): 74.1 vs 75.8 Sex (female): 60.3 % vs 47.7 % Patients: High risk elderly patients with chronic diseases Setting: primary care	Elderly patients with chronic diseases congestive heart failure, hypertension, diabetes, dementia, depression	*Guided Care:* Trained registered nurses working in primary care practices, in close collaboration with PCPs (1 nurse per 2 PCPs): - assess patient and caregiver needs; - develop an individualized care plan; - promote patient self-management; - monitor patient’s condition; - coordinate transitions between healthcare services; - facilitate access to community resources.	Lower insurance expenditures (*p* = 0.35)
Gravelle, 2007/UK [60]	NRS	Sample size: 64 intervention primary care practices Age: not reported. Patients: Elderly patients (≥65 years old) and a history of emergency admission Setting: primary care	Elderly patients at high risk of emergency admission	*Case management:* - development of individualized care plan by the nurse practitioners in collaboration with PCP; - coordination of services to prevent fragmentation of services; - arrangement of access to community-based services.	
Horwitz, 2005/USA [40]	RCT	Sample size: 230 (121 vs 109) Age (mean): 51.2 % vs 50.5 % (less than 30 years old) Patients: Uninsured population Setting: primary care	Uninsured patients (except substance abuse and mental health issues)	*The Community Access Program:* - identification of uninsured patients before discharge from the hospital who don’t have a PCP; - assistance with enrolment to one of four PCPs; - faxing the patient data to a case managers of the primary care facility; - case managers contacted the patients to arrange an appointment.	Reduction in average cost of an emergency room visit
Palfrey, 2002/USA [32]	NRS	Sample size: 267 (150 vs 117) Age (0–5): 56 % vs 55.6 % Sex (female): 33.3 % vs 33.3 % Patients: Children with special health care needs Setting: primary care	Children with special health care needs	*Pediatric Medical Home:* - designation of a pediatric nurse practitioner (PNP); - designation of a lead PCP; - arrangement of the schedule for the PNP (8 h per week devoted to the management of children with special needs) by the lead physician; - in-home follow-up by the PNP; - assistance with appointments and medication supply; - development of the individualized health plan; - sharing of the health plan and evolution of the condition with specialists; - participation of a local parent consultant.	
Farmer, 2005/USA [27]	NRS	Sample size: 102 (51 vs 51) Age: 7.4 ± 5.1 Participants: Children with special health care needs Setting: primary care	Children with special health care needs (mental and neurological disorders, congenital anomalies)	*Medical home:* - delivery of care by PCP, nurse practitioner, a parent consultant; - nurse practitioner provides: a home visit to conduct comprehensive assessment of medical and non-medical needs, a personalized letter describing health and services available to meet these needs, an individualized health plan for the child, at least 1 follow-up; - nurse practitioner acted as consultant for 3 primary care practices; - nurse practitioner interacts regularly with referring physicians and a designated nurse at each primary care practice; - medical care was provided by these practices; - a web-site was developed to ease access to additional supports and recourses by families and physicians.	
Druss, 2001/USA [36]	RCT	Sample size: 120 (59 vs 61) Age (mean): 45.7 ± 8.4 vs 44.8 ± 8.0 Sex (female): 0 % vs 1.6 % Patients: Patients with mental disorders Setting: primary care	Patients with mental disorders: schizophrenia, posttraumatic stress disorder, major affective disorder, substance abuse	*Integrated care:* Integrated mental health service in the primary care (a multidisciplinary team of a nurse practitioner, PCP, a nurse case manager, physicians in the psychiatry and mental health clinics): - supervision of the nurse practitioner (providing basic medical care) by the primary care provider; - primary care provider is a liaison of primary and specialized services; - the nurse provides education, preventive services, follow-up (telephone, e-mail, face-to-face), schedules an appointment; - the nurse practitioner serves as a liaison of 3 mental health teams.	
Counsell, 2007/USA [38]	RCT	Sample size: 951 (474 vs 477) Age (mean): 71.8 ± 5.6 vs 71.6 ± 5.8 Sex (female): 75.5 % vs 76.5 % Patients: Low-income seniors Setting: primary care	Low-income seniors (less than 200 % of the Federal Level of Poverty) with geriatric conditions such as difficulty walking, falls, pain, urinary incontinence, depression, vision and hearing problems, dementia	*Geriatric Resources for Assessment and Care of Elders (GRACE):* - in-home comprehensive geriatric assessment by a nurse/social worker; - development of individualized care plan by a multidisciplinary team (a geriatrician, pharmacist, physical therapist, mental health social worker, community-based services representatives); - regular meeting of the multidisciplinary team and PCP; - ongoing support via en electronic medical records and web-based tracking system.	
Landi, 2001/Italy [63]	NRS	Sample size: 1204 (before-after) Age (mean): 77.4 ± 9.7 Sex (female): 58.5 % Patients: Frail older people Setting: primary care	Frail older people	*Home care program:* - development of the community Geriatric Evaluation Unit (“a single enter center”) consisting of a geriatrician, a social worker, a physiotherapist, nurses jointly with a PCP; - initial and follow-up assessments by case manager (a nurse); - coordination of services delivery; - facilitation of access to community-based services; - PCP involved directly in care planning, case finding, and emergency situations.	27 % cost reduction with an estimated saving of $1,200 for each patient
Callahan, 2006/USA [51]	RCT	Sample size: 153 (84 vs 69) Age (mean): 77.4 ± 5.9 vs 77.7 ± 5.7 Sex (female): 46.4 % vs 39.1 % Patients: dementia patients Setting: primary care	Patients with dementia living in the community	*Collaborative care model:* - development of individualized care plan for the patient-caregiver dyad; - regular assessment of patients’ condition; - medication management by PCP; - weekly review of care and adherence to guidelines by multidisciplinary team (geriatric nurse practitioner, PCP, geriatrician, geriatric psychiatrist, psychologist) - monitoring of health condition and communication of healthcare professionals via web-based system.	$1000 annual cost of the case manager per patient (75 patients per year)
Continuity of care via arrangement for follow-up					
Sin, 2004/Canada [57]	NRS	Sample size: 125 (63 vs 62) Age: 22.5 ± 13.7 vs 22.7 ± 12.6 Sex (female): 46 % vs 74 % Patients: Patients with asthma Setting: primary care	Patients with asthma	*Enhanced care:* - follow-up appointment with PCP within 4 weeks of discharge; - a study coordinator makes an appointment on behalf of the patient; - in case a patient does not have a PCP, he is offered to choose from a list of physicians willing to accept new patients; - a reminder telephone call 1 or 2 days before the scheduled follow-up visit;	
DeHaven, 2012/ USA [48]	NRS	Sample size: 574 (265 vs 309) Age: 35.7 ± 12 vs 35 ± 12.1 Participants: Uninsured adults Setting: primary care	Uninsured low-income working individuals	*Project Access Dallas:* - monthly meeting with a community health worker; - patients assigned to a PCP; - referral to the specialist if needed; - pharmacy benefits ($750 a year); - PCPs and specialists donated their services depending on their capacity	The intervention resulted in less direct (*p* < 0.01) and indirect costs (*p* < 0.01).
Institution incentives^a^					
Addink, 2011/UK [58]	NRS	Sample size: 24 practices in three local primary care trusts Age: not reported. Participants: Ethnic minority. Setting: primary care	Patients from ethnic minority groups (non-white ethnicity)	*Pay for performance scheme:* Primary care practices received payment according to their performance based on the reporting of their patients.	- £36 million received for participation; - £72 million received based on the positive responses of patients (£1.37 per highly satisfied registered patient).
Tan, 2012/New Zealand [66]	Mixed methods study (convergent parallel design)	Sample size: the whole population Age: not reported. Patients: Ethnic and refugee communities, young people Setting: primary care	Prioritized population: high deprivation, Maori, Pacific communities, refugees, young people	*Primary care framework:* Sustained and targeted investments over five years in: - development of service delivery for equitable access (community health workers, additional nurses and outreach services, youth service); - engagement of healthcare professionals to develop these services; - development of health approaches in collaboration with ethnic groups (e.g., iwi); - information sharing across the range of support services; - building on intersectoral relationships; - promotion of preventive programs (e.g., increase of physical activity); - support of leadership by clinicians in more community-based care.	$6 M of annual funding over five years
Feinglass, 2014/USA [28]	NRS	Sample size: 293 (138 vs 158) Age (45–64): 48.8 % vs 58.8 % Sex (female): 68 % vs 60 % Participants: Uninsured adults Setting: primary care	Uninsured adults with a household income below 200 % of Federal Poverty Level.	*County Health Care program (Access DuPage):* - assigns patients to PCPs; - pays a small capitated fee to primary care clinics and PCPs while most of funding comes from county hospitals, county government, and foundations; - coordinates purchase of medications with small enrollee copays; - handles applications for Drug Assistance Programs which provides enrollees with medications.	Decrease of amount of payment/copayment for a visit (*p* < 0.0001).
Capitation^a^					
Davidoff, 2008/USA [25, 67]	NRS	Sample size: 574 (265 vs 309) Age: 2–17 Participants: Children with chronic health conditions Setting: primary care	Children with common chronic health conditions such as attention deficit disorder, mental retardation, Down syndrome, asthma, cerebral palsy, sickle cell anemia, muscular dystrophy, autism, congenital or other heart diseases, diabetes.	*Primary care case management:* - PCPs are paid for care coordination to serve as “gatekeeper” for referrals to specialty services; - care provided by PCPs is focused on early intervention, appropriateness, and coordination.	

*RCT* Randomized Controlled Trial, *NRS* Non-Randomized Study, *NS* Non-significant

^a^ Financial interventions according to the EPOC classification

**Table 2:** Vulnerability context

Vulnerability context	Included studies, n (%)
Socioeconomically disadvantaged (*n* = 14)	
Uninsured	11 (28 %)
Immigrants	1 (2 %)
Formerly incarcerated	2 (5 %)
Racial/ethnic minority (*n* = 1)	1 (2 %)
First Nations (Maori, Alaska Native, American Indian, Pacific) (*n* = 4)	4 (10 %)
Chronic diseases (*n* = 25)	
Multi-morbidity (chronic heart failure, chronic obstructive pulmonary diseases, hypertension, dyslipidemia, diabetes, obesity)	5 (13 %)
Multi-morbidity non-specified (e.g., functional decline, frailty)	5 (13 %)
Geriatric conditions (difficulty walking/falls, urinary incontinence, vision/hearing problems, dementia)	(5 %)
Mental diseases (chronic psychosis, depression, anxiety, bipolar disorder, schizophrenia, personality disorders, panic disorder)	5 (13 %)
Diabetes	2 (5 %)
Asthma	1 (2 %)
HIV	2 (5 %)
Congenital conditions (mental retardation, Down syndrome, cerebral palsy, muscular dystrophy, autism)	3 (8 %)
Elderly with chronic diseases (*n* = 11)	11 (28 %)
Children with chronic diseases (*n* = 5)	5 (13 %)

Thirty-five studies (89.7 %) concerned organizational interventions, including revision of professional roles [61, 62], clinical multidisciplinary teams [44, 45, 50, 52, 54], formal integration of services [26, 29, 31, 33, 34, 41, 46, 53, 56, 64, 65], and continuity of care via case management [27, 30, 32, 35–40, 43, 47, 49, 51, 55, 60, 63] or arrangements for follow-up [48, 57]. Four studies (10.3 %) concerned financial interventions, namely institution incentives [28, 58, 66] and capitation [25].

### Description of the organizational interventions

#### Continuity of care via case management

This organizational intervention is designed to coordinate different medical and social services via a case manager (i.e., a nurse) who closely works with PCP [27, 30, 32, 35–40, 43, 47, 49, 51, 55, 60, 63]. A case manager is responsible for assessment of care needs, development of care plan in collaboration with other health care professionals, regular follow-up and liaison of services. In the identified studies majority of participants were elderly patients with multiple chronic conditions and functional disabilities [35, 38, 39, 49, 51, 55, 59, 60, 63]. Other categories of the patients were uninsured [30, 40, 47] and formerly incarcerated people [37, 43], children with special health care needs [27, 32], and patients with psychiatric disorders [36].

#### Formal integration of services

This organizational intervention targets to bring all services (medical and social) at one point [44, 45, 50, 52, 54]. Four types of intervention strategies have been used in the identified studies. The first strategy was to bring together primary care and secondary/tertiary services, i.e., integrate specialists into primary care settings such as mental health teams [29, 41, 42, 65], community service teams [26], and alcohol-substance abuse counselors [31]. The second strategy was for brokers or community health workers to identify proactively eligible patients (e.g., in the emergency room) and assign them to a primary care practitioner [31, 33, 34, 46]. Third, a network was developed and integrated services using a ‘single entry point’ (integration of home care, rehabilitation and hospital services) with 24/7 telephone access [53, 64]. Fourth, informatics-based integration allowed virtual monitoring of complex health conditions from primary care to hospital-based services (telehomecare) [56]. The main categories of vulnerable populations targeted by this intervention were patients with low income or uninsured [26, 31, 33, 34, 46], patients with mental health problems [29, 41, 65], and elderly patients with multiple chronic conditions [53, 56].

#### Clinical multidisciplinary team

This organizational intervention is based on two approaches - creation of a team with healthcare professionals from multiple disciplines [45, 50] or addition of a new member to the existing team (i.e., local indigenous health workers [62] or ethnic group representative [44], a dietitian [52], a nurse practitioner in a team of mental health professionals [54]).

#### Continuity of care via arrangement for follow-up

This organizational intervention is based on close follow-up either post discharge [57] or on a predefined frequency [48] to ensure timely access to services.

#### Revision of professional role

A new role has been assigned to provide a different care in one study (a social worker of local ethnicity trained in skin infection recognition) [61].

#### Institution incentive and capitation

These organizational interventions are based on financial incentives to provide a financial reward for performing specific action [28, 58, 66] or to award a certain amount per patient seen [25, 67].

### Pattern ‘dimension-outcome’

Regarding access dimensions and outcomes, the characteristics of studies on local/regional interventions and state/national interventions are described in Appendix 2 and 3, respectively. The dimension-outcome patterns are summarized in Table 3. The pattern analysis revealed one pattern. Results of the 10 studies on interventions classified as ‘Formal integration of services’ showed that in almost all cases these interventions were associated with three dimensions of access (approachability, availability and affordability) and reduction of hospitalizations (four/four studies), emergency department admissions (six/six studies), and unmet healthcare needs (five/six studies) (number of studies with a positive outcome/number of studies assessing this outcome). Various research designs were used: non-randomized (*n* = 7), randomized controlled (*n* = 1), quantitative descriptive (*n* = 1), and mixed methods (*n* = 1). These 10 studies were reported across 12 papers (Table 1) [26, 29, 31, 33, 34, 41, 42, 46, 53, 56, 64, 65].

**Table 3:** Pattern dimension-outcome

Organizational intervention	Number of studies	Outcomes^a^			Pattern ‘Dimension- Outcome’
		↓HR	↓ ED admission	↓ Unmet health care needs	
Continuity of care via case management	16^b^	8/15	7/13	3/3	No
Formal integration of services	10^c^	4/4	6/6	5/6	Yes^d^
Clinical multidisciplinary teams	6	1/1	0/4	2/2	No
Continuity of care via arrangement for follow-up	2	1/1	1/1	-	No
Revision of professional roles	1	-	-	1/1	No
Institution incentives	3	1/2	0/1	1/2	No
Capitation	1	-	1/1	0/1	No

*HR* hospitalisation rate, *ED* emergency department, *RCT* Randomized Controlled Trial, *NRS* Non-Randomized Study

^a^Numerator: Number of studies with a positive outcome; Denominator: Number of studies assessing the outcome

^b^9 RCTs and 7 NRSs

^c^7 NRS, 1 RCT, 1 quantitative descriptive and 1 mixed methods study

^d^Associated with three dimensions of access: approachability, availability and affordability

A possible subpattern has been found in the category of organizational interventions "Continuity of care via case management": reduction of unmet health care needs in the studies associated with two dimensions of access - approachability and availability [27, 32, 36]. Non-randomized (*n* = 2) and randomized controlled (*n* = 1) designs were used. However, considering a limited number of identified studies a conclusion on the 'dimension-outcome' pattern cannot be made.

## Discussion

This scoping review included 39 studies of organizational interventions aimed at improving access to primary care for vulnerable populations (patients with chronic conditions and socioeconomically disadvantaged people), which have evaluated the impact of these interventions on hospitalization, emergency department admission, or unmet health care needs. Results revealed one ‘dimension-outcome’ pattern: the formal integration of services in which the reduction of hospitalization, emergency department admission and unmet health care needs was associated with three dimensions of access (approachability, availability and affordability), specifically for patients with low income or uninsured, patients with mental health problems, and elderly patients with multiple chronic conditions.

Formal integration of services means bringing all primary medical and social service providers together, typically with mental health service professionals, to meet the needs of the disadvantaged population. This is similar to ‘seamless care’ in inter-professional education (transversal integration) and inter-organizational pharmaceutical care (vertical integration) where students from multiple health disciplines, hospital and community pharmacists, formally do teamwork together, respectively [68, 69]. This also refers to ‘shared care’ or ‘collaborative care’ in mental health for instance, which consists of “a structured system for achieving integration of care across multiple autonomous providers and services with both primary and secondary care practitioners contributing to elements of a patient’s overall package of care” where “mental health experts work with first-line care providers in the delivery of mental health promotion, illness prevention, detection and treatment of mental illnesses, as well as rehabilitation and recovery support [70, 71].” For example, in the identified intervention studies formal integration was mainly done through teams including both primary care physicians and specialized health service providers in mental health, alcohol and substance abuse, and home care programs. An illustration of formal integration is a medical home (called Family Medicine Groups in Quebec or Family Health Teams in Ontario, Canada) when parents in situation of vulnerability are informed about all available services (approachability) to get them in time (availability) and free (affordability) including transcultural child mental health support if needed [72].

Our results indicate that the most commonly evaluated dimensions of access were approachability, availability and affordability for interventions targeting vulnerable populations. The most commonly evaluated type of intervention was continuity of care via case management; this type was not associated with a reduction of hospitalization and emergency department admission, although we did find in a recent systematic review that this type of intervention is effective for elderly patients with dementia [16, 17].

In addition, our results suggest a research gap in looking at vulnerability and access to primary care services from a patient perspective. Specifically, a paucity of research regarding reduction of hospitalization, emergency department admission, and unmet service needs outcomes with regard to five types of intervention (clinical multidisciplinary teams, revision of professional role, continuity of care via arrangement for follow-up, institution incentive, and capitation). Moreover, this work suggests a need for more research on these outcomes as well as the acceptability and appropriateness dimensions of access.

Ultimately, patients’ and caregivers’ ability to identify healthcare needs, and to know where to access primary care, as well as the ability to engage with care in order to receive what is actually appropriate could be the crucial gaps in access for vulnerable populations in certain contexts. This illustrates the challenge of embedding patient’s self-efficacy in policies. This scoping review suggests it can be easier to target structural resources and clinical behaviors (supply-side perspective) to adapt services to the needs, expectations and abilities of patients, rather than to empower patients and caregivers to more broadly engage in care access, which is what the Australian-Canadian IMPACT program is seeking to accomplish.

While only one ‘dimension-outcome’ pattern was found in this review, the limited number of included studies on patients in situation of vulnerability and the theory-driven approach may have precluded finding others. For example, we found few studies on the three outcomes of interest outside the formal integration of services and case management. This might reflect the fact that few innovations have been evaluated or published yet. For instance, several organizational innovations have been put in place in OECD countries to improve access to primary care (i.e., advanced access) [73]. Although numerous articles describe primary care organizational innovations for vulnerable populations, few report on the evaluation of these innovations. While three main databases were searched, subsequent systematic review may include an exhaustive search of evidence in multiple databases (including management databases, e.g., Health Business Elite), the grey literature, and citation tracking (e.g., in Scopus) with selection of bibliographic records and full-texts by two independent reviewers. However, the broad criteria of our search make it less likely that important articles were missed. Another implication for future review is derived from the focus of this scoping review on three outcomes and the limitation of the EPOC classification. Studies on other outcomes (i.e., health status) were excluded, while they can be considered. The EPOC classification of interventions pointed to key components of interventions, while other components can be considered. Various intervention elements appeared across different EPOC categories, suggesting the need for an inductive and finer grained typology of interventions to inform future practice. For example, the above-mentioned IMPACT program, led by the last four co-authors, is developing an inductive taxonomy of organizational interventions for improving access to primary care for vulnerable populations. This taxonomy could be useful for planning future research and reviews, improving practice and developing policies.

## Conclusion

While there appears to be a limited number of published research studies about organizational interventions aimed at improving access to primary care for vulnerable populations, our scoping review showed that there are enough studies for a future systematic review to test the following hypothesis: formal integration of services (increased approachability, availability and affordability of primary care services) could be associated with a reduction of hospitalization, emergency room admission and unmet health care needs. Not surprisingly, our results also suggest approachability, availability and affordability could play an important role in access to care for vulnerable populations. Considering that this scoping review included all types of evidence, and suggests access to primary health care services for vulnerable populations could be improved by formal integration of services, future research can provide stronger evidence on finer grained types of interventions and other types of outcome.

## Appendix 1

**Example of search strategy in Embase database**

1. translational research/(5528)
2. integrated health care system/(6588)
3. Case Management/(7314)
4. Knowledge Management/(762)
5. exp quality control/(220922)
6. health care quality/(153298)
7. Organi?ational innovation*.tw. (71)
8. innovat*.ti. (14842)
9. Organi?ational change*.tw. (1807)
10. organi?ational model*.tw. (548)
11. (diffusion adj2 innovation*).tw. (504)
12. Integrated delivery system*.tw. (569)
13. Integrated Health Care System*.tw. (325)
14. Integrated Health* System*.tw. (544)
15. (program or programs or programme or programmes).tw. (468596)
16. medical care team*.tw. (56)
17. interdisciplinary health team*.tw. (11)
18. healthcare team*.tw. (1537)
19. health care team*.tw. (2704)
20. case management.tw. (7055)
21. managed care.tw. (15501)
22. knowledge management.tw. (838)
23. healthcare quality.tw. (1068)
24. health care quality.tw. (1756)
25. quality of health care.tw. (4484)
26. quality of healthcare.tw. (1334)
27. quality management.tw. (5531)
28. quality assurance.tw. (17475)
29. case coordination.tw. (25)
30. (intervention or interventions).tw. (582350)
31. multidisciplinary team*.tw. (12026)
32. or/1-31 (1321868)
33. health care access/(34233)
34. health care availability/(7450)
35. Healthcare Disparity/(5959)
36. exp patient attitude/(217912)
37. (availab* adj2 (healthcare or health care or health service*)).tw. (1393)
38. (access* adj2 (healthcare or health care or health service*)).tw. (9122)
39. access*.ti. (40638)
40. program* accessibility.tw. (20)
41. program* availability.tw. (62)
42. affordability.tw. (2214)
43. approachability.tw. (103)
44. appropriateness.tw. (13616)
45. or/33-44 (311948)
46. 32 and 45 (86444)
47. exp child health care/(47406)
48. exp community care/(63087)
49. Mental Health Services/(31280)
50. exp Primary Health Care/(86682)
51. General Practice/(43336)
52. child health service*.tw. (444)
53. infant health service*.tw. (16)
54. community nurs*.tw. (1833)
55. community mental health service*.tw. (588)
56. community health service*.tw. (551)
57. community pharmac* service*.tw. (123)
58. maternal health service*.tw. (246)
59. preventive health service*.tw. (361)
60. (senior center* or senior centre*).tw. (462)
61. (center* for the aged or centre* for the aged).tw. (334)
62. primary care.tw. (76389)
63. primary health care.tw. (11687)
64. primary healthcare.tw. (2676)
65. general practice.tw. (21225)
66. family practice.tw. (3694)
67. family medicine.tw. (6162)
68. or/47-67 (285192)
69. 46 and 68 (15001)
70. Vulnerable Population/(6013)
71. Poverty/(23599)
72. Unemployment/(7459)
73. Homelessness/(5938)
74. chronic disease/(97466)
75. mental disease/(118256)
76. exp *mental disease/(664947)
77. exp *aged/(16069)
78. frail elderly/(5142)
79. very elderly/(9994)
80. Minority Group/(8274)
81. Disabled Person/(12980)
82. exp "Drug Use"/(150974)
83. medically uninsured/(315)
84. illegal immigrant/(131)
85. immigrant/(8277)
86. indigent/(329)
87. lowest income grouup/(0)
88. medically underserved/(262)
89. refugee/(5352)
90. exp Terminally Ill patient/(4703)
91. vulnerab*.tw. (76118)
92. poverty.tw. (13629)
93. high risk population*.tw. (8227)
94. high risk patient*.tw. (28251)
95. complex patient*.tw. (1884)
96. complex need*.tw. (851)
97. sensitive population*.tw. (315)
98. disadvantaged.tw. (6913)
99. (underserved or under served).tw. (5638)
100. indigen*.tw. (18928)
101. tribes.tw. (2017)
102. native*.tw. (124764)
103. aboriginal*.tw. (5424)
104. low income.tw. (17600)
105. unemploy*.tw. (10051)
106. underemploy*.tw. (179)
107. homeless*.tw. (5781)
108. (street people or street person*).tw. (12)
109. social* stigma*.tw. (1141)
110. social* isolat*.tw. (4447)
111. inequalit*.tw. (13899)
112. uninsured.tw. (5190)
113. underinsured.tw. (459)
114. uneducated.tw. (402)
115. low educat*.tw. (4366)
116. poorly educated.tw. (260)
117. illitera*.tw. (3098)
118. chronic disease*.tw. (39799)
119. chronic* ill*.tw. (13506)
120. chronic condition*.tw. (10227)
121. aged.tw. (351635)
122. old.tw. (694632)
123. older.tw. (266367)
124. elderly.tw. (162147)
125. frail*.tw. (10361)
126. (senior or seniors).tw. (24681)
127. functional* impair*.tw. (12914)
128. disabled.tw. (13707)
129. disability.tw. (95888)
130. disabilities.tw. (28088)
131. handicapped.tw. (2633)
132. physically challenged.tw. (54)
133. mentally challenged.tw. (89)
134. mental disorder*.tw. (23602)
135. mental* ill*.tw. (21844)
136. psychiatric diagnos*.tw. (6839)
137. drug use*.tw. (42628)
138. drug abuse*.tw. (12151)
139. drug addict*.tw. (6598)
140. drug dependen*.tw. (4120)
141. drug habit*.tw. (69)
142. "substance use".tw. (19197)
143. substance dependen*.tw. (2410)
144. substance addict*.tw. (285)
145. uninsured.tw. (5190)
146. underinsured.tw. (459)
147. terminal* ill*.tw. (4662)
148. minority.tw. (38757)
149. minorities.tw. (6698)
150. immigra*.tw. (19128)
151. foreigner*.tw. (952)
152. refugee*.tw. (4336)
153. or/70-152 (2572418)
154. 69 and 153 (7631)
155. limit 154 to yr = "2000 -Current" (7118)
156. limit 155 to (english or french) (6891)
157. qualitative research*.mp. (29502)
158. qualitative stud*.mp. (18918)
159. action research.mp. (2607)
160. Participatory Research/(1916)
161. participatory research.mp. (3107)
162. case stud*.mp. (68678)
163. ethno*.mp. (67315)
164. grounded theory.mp. (6560)
165. phenomeno*.mp. (137337)
166. Narrative/(2274)
167. narrative*.mp. (16468)
168. biograph*.mp. (4728)
169. Autobiograph*.mp. (3202)
170. documentar*.mp. (1411)
171. qualitative synthes*.mp. (229)
172. active feedback.mp. (106)
173. conversation*.mp. (11040)
174. discourse*.mp. (9173)
175. thematic.mp. (10376)
176. qualitative data.mp. (6953)
177. key informant*.mp. (3676)
178. focus group*.mp. (25043)
179. case report*.mp. (1137960)
180. exp Interview/(123867)
181. interview*.mp. (240740)
182. exp Observational method/(1826)
183. observer*.mp. (52777)
184. visual data.mp. (249)
185. (audio adj record*).mp. (2581)
186. Cultural Anthropology/(32392)
187. experience*.mp. (670612)
188. or/157-187 (2271697)
189. exp clinical study/(4260567)
190. exp Methodology/(2712060)
191. randomization/(54544)
192. Placebos/(185259)
193. Crossover procedure/(35159)
194. or/189-193 (5940545)
195. (clinic* adj25 trial*).mp. (966368)
196. random*.mp. (862922)
197. control*.mp. (5237933)
198. (latin adj square).mp. (2318)
199. placebo*.mp. (243987)
200. or/195-199 (5877623)
201. exp Comparative Study/(666277)
202. comparative stud*.mp. (469566)
203. Validation Study/(44621)
204. validation stud*.mp. (50401)
205. evaluation research/(1614)
206. evaluation stud*.mp. (5068)
207. Follow-Up/(685736)
208. followup.mp. (21000)
209. follow-up.mp. (902021)
210. cross over.mp. (14288)
211. crossover.mp. (50503)
212. prospective*.mp. (548205)
213. volunteer*.mp. (132028)
214. or/201-213 (2089573)
215. singl*.mp. (1081892)
216. doubl*.mp. (392712)
217. trebl*.mp. (253)
218. tripl*.mp. (76252)
219. or/215-218 (1456962)
220. mask*.mp. (51854)
221. blind*.mp. (236161)
222. 220 or 221 (284003)
223. 219 and 222 (164051)
224. 194 or 200 or 214 or 223 (9418632)
225. exp Health Survey/(140568)
226. Health Care Survey/(5933)
227. exp Risk/(1311750)
228. exp Incidence/(231106)
229. exp Prevalence/(360569)
230. exp Mortality/(512165)
231. cohort*.mp. (395294)
232. case-control.mp. (122486)
233. cross sectional.mp. (214289)
234. (health* adj2 survey*).mp. (155311)
235. risk.mp. (1871315)
236. incidence.mp. (563598)
237. prevalence.mp. (512194)
238. mortality.tw. (486678)
239. "case series".mp. (43403)
240. "time series".mp. (24993)
241. "before and after".mp. (171898)
242. prognos*.mp. (514923)
243. predict*.mp. (1017431)
244. course*.mp. (614624)
245. or/225-244 (4558876)
246. (mixed adj5 method*).mp. (9599)
247. multimethod*.mp. (1003)
248. (multiple adj5 method*).mp. (22707)
249. or/246-248 (33153)
250. qualitative.mp. (135948)
251. Qualitative Research/(23741)
252. quantitative.mp. (442547)
253. or 251 (135948)
254. 252 and 253 (48559)
255. 249 or 254 (79725)
256. 188 or 224 or 245 or 255 (10841719)
257. 256 not (letter/or editorial/) (10370545)
258. 257 not (animal not human).sh. (10070426)
259. 156 and 258 (6064)
260. limit 259 to embase (4439)

## Appendix 2

**Table 4:** Characteristics of included studies (local/regional intervention): Access dimension and outcome

Study ID	Dimensions of access of primary care services					Dimensions of ability of consumers	Outcomes		
	Approachability	Acceptability	Availability and Accommodation	Affordability	Appropriateness	Ability to (1) Perceive; (2) Seek; (3) Reach; (4) Pay; (5) Engage	Avoidable hospitalization	Avoidable ED admission	Unmet health care needs
Revision of professional roles									
Gray, 2013 [61]	Students with symptoms of sore throat and skin infection were regularly searched	The social worker providing health care services was the same ethnic group	Health care services delivered directly at primary school, at home (for household members), regular phone contacts.	Free health care program		*Ability to perceive:* education of parents on the importance of the provided services; *Ability to reach:* availability of the health care program in the primary school. *Ability to pay:* free health care program			Health care service received in a timely manner.
Clinical multidisciplinary teams									
Doey, 2008 [54]			“One-stop shopping for clients” – co-location of primary health services with mental care.	Intervention in public health care system	Timeliness of primary health services delivery (preventive measures)		75 % decrease of hospitalization	- 51.6 % decrease in the number of emergency visits; - 38 % never used emergency services.	
Crustolo, 2005 [52]	Referral to the dietitian by the PCP if nutrition-related problems were present		Location of dietitian in primary care.	Intervention in public health care system	Intervention was offered at an early stage of the health condition (e.g., priority to prevent childhood obesity).				Patients were satisfied with: - length of wait for appointment; - getting through by phone; - length of time waiting; - time spent with healthcare professional; - explanation of what was done; - personal manner of healthcare professional; - major health concerns were addressed.
McCuloch, 2000 [45]	Patients identified through diabetes registers		Available assessment by specialists in primary care practice		Timely assessment of patients to avoid complications (retinal screening, screening for microalbuminuria, hyperglycemia)		Decrease by 17 %	No difference	
Michelen, 2006 [44]	Information about frequent users of ED (3 or more times in the past 6 months) was e-mailed to healthcare professionals who contacted them thereafter.	Healthcare professionals providing the intervention were from the same ethnical background	Patients living in three neighborhoods (Harlem, Washington Heights, Inwood) were enrolled			*Ability to reach:* primary care services available in the geographic catch area (neighborhood) of the intervention.		Decrease at 3 months (*p* = 0.002), no difference at 6 month	
Driscoll, 2013 [50]				Payers are Indian Health Services, Medicaid/Medicare, independent insurers				Decrease (*p* < 0.001)	
Formal integration of services									
Day, 2006 [65]			Location of specialized mental health services in primary care practice.						Satisfied with: - length of wait prior to the first appointment (85.1 %); - location (95.5 %); - quality of venue (89.5 %); - duration of the appointment (92.7 %); - 94 % found the appointment convenient.
Garg, 2012 [26]					Community services were provided appropriate to the needs (e.g., employment to unemployed participants).				Reduction of unmet social needs (50 % of families enrolled in at least one community-based resources).
Lamothe, 2006 [56]			The monitoring of health condition was from home of participants.		Timely delivery of services based on the alerts received from patients.	*Ability to engage:* participation of patients and healthcare professionals in decision-making regarding of the treatment options based on the measurements of vital signs.		Decrease in the number of emergency visits	- no need to travel to physician’s office for blood pressure reading; - absence of waiting time to have blood pressure read by a nurse; - better access to services and easier access to nursing and medical expertise.
Tourigny, 2004; Hebert, 2010 [53, 64]	24/7 access to the Health Info Line for the assessment of needs.		“The single entry point” mechanism for accessing the services in the area for frail seniors with complex needs.	Intervention in public health care system	The continuous nature of the intervention (close collaborative work of PCP, case manager, and multidisciplinary team).		- increase of hospitalization within 10 days (*p* = 0.043); - no difference within 30 or 90 days; - higher in year 1 (31 % vs 28 %, *p* = 0.281); - no difference over 4 years (*p* = 0.113).	- no difference within 10 days; - higher in year 1 and 2 (*p* < 0.001); - no difference in year 3 and 4.	
Levkoff, 2004; Chen, 2006 [41, 42]	Referral to the mental health services based on the screening by primary care providers		Co-location of primary care services with mental health services						- got the service patients wanted (*p* = 0.01); - service received met patients needs (*p* = 0.0001).
Brown, 2005 [29]	Referral to the mental health services by the PCP; identification in the database patients with a large number of hospitalizations.		Co-location of primary care services with mental health services				Decrease (*p* = 0.02)	Decrease (*p* = 0.05)	
MacKinney, 2013 [33]	Contact of identified people without insurance by the county social worker to offer an access to primary health services			Absence of co-payment for basic medical services			Decrease (13 % vs 6 %; *p* < 0.03)	Decrease (32 % vs 19 %; *p* < 0.0001)	
Bradley, 2012 [34]	Contact of identified people without insurance to offer an access to primary health services		Primary care providers located near the residence of patients	Absence of payment for primary care services			Decrease (*p* < 0.01)	Decrease (*p* < 0.01)	
Kaufman, 2000 [31]	Uninsured patients according the eligibility criteria were enrolled.		The program was eligible for the residents of New Mexico county only.	Small copayment depending on the poverty level (ranged from no premium to $10 per patient per month)		*Ability to pay:* ranged from no payment to a small monthly premium.	Decrease (*p* < 0.0001)	Decrease (*p* < 0.0001)	Decrease of time for the first appointment with PCP (from 45 to 28 days).
Roby, 2010 [46]	Uninsured patients were enrolled at the time they sought for health services.							Decrease (*p* < 0.05)	
*Continuity of care via case management*									
Beland, 2006 [55]	Intervention delivered through the public community organizations responsible for home care		The intervention team physically was located in the public community organizations	Intervention in public health care system			50 % reduction in the number of “bed-blockers” (*p* < 0.05) but no overall effect on hospitalization.	Trend for 10 % lower utilization (*p* = NS)	
Glendenning-Napoli, 2012 [30]	Uninsured patients with frequent hospital and emergency use were contacted by the phone to enroll in the program.					*Ability to perceive:* assessment of patient health literacy level and ability to manage health condition is a part of the intervention; *Ability to engage:* participants were involved in the development of the preventive care plan tailored to their needs.	Decrease (*p* < 0.0001)	Decrease (*p* < 0.0007)	
Leff, 2009; Boult, 2011 [35, 59]	Insured older patients at high risk of health service use were contacted (screening based on the insurance claims)		In-home assessment of needs	Eligible patients were those with existing insurance.		*Ability to reach:* some elements of the intervention were delivered at home (e.g., assessment of needs) *Ability to engage:* involvement in the development of individual care plan.	Decrease (*p* = NS)	Decrease (*p* = NS)	
Shah, 2011 [47]	Uninsured frequent ED users were identified and enrolled					*Ability to reach:* follow-up at home and during hospitalization; *Ability to engage:* involvement in the development of the individual care plan.	Decrease (*p* < 0.0001)	Decrease (*p* < 0.0001)	
Wang, 2012 [43]	Mandatory attendance of an appointment with a community health worker within 2 weeks of the release date from the prison	Health care services were provided and coordinated by a healthcare professional with a history of incarceration.					No difference (*p* = 0.34)	Decrease (*p* < 0.04)	
Wohl, 2011 [37]	Services were offered to HIV patients prior their release from the prison		Services were identified in the neighborhood		Individualized care plans were developed according to the needs	*Ability to engage:* clients were actively involved in the development of care plan for short and long-term objectives.	No difference	No difference	
Sylvia, 2008 [39]	Patients referred by PCPs				Individualized care plans were developed according to the needs	*Ability to engage:* patients were actively involved in the development of care plan.	Decrease (*p* = 0.19)	Decrease (*p* = 0.20)	
Horwitz, 2005 [40]	Patients identified at discharge		Intervention was offered to patients living in the proximity of primary care facilitates				No difference	No difference	
Palfrey, 2002; 2004 [32]	Children already receiving care in pediatric primary care practices were approached		- In-home assessment of needs and regular home follow-up; - Intervention was designed for the residents of the particular neighborhood.			*Ability to reach:* some elements of the intervention were delivered at home (e.g., assessment of needs); *Ability to engage:* involvement in the development of the individual care plan; participation of a local parent consultant in the development and amendment of the intervention.	Decrease (*p* < 0.01)	No difference	Decrease of unmet health care needs: - getting a phone calls returned (61 %); - getting an appointment (60.9 %); - getting early medical care (61.4 %); - getting resources for a child (59.7 %); - getting letters of medical necessity (66.9 %); - communicating with a child’s doctor (60.9 %); - getting referral to doctors (60.5 %).
Farmer, 2005 [27]	Patients referred to the program according to the eligibility criteria		Children residing in the region primary care clinics provide health services for.	Participants were enrolled in Medicaid fee-for-service, Medicaid managed care, and commercial health insurance		*Ability to perceive:* children were already involved in the services provided by multiple medical specialists due to severe health disorder/s interfering their everyday functioning; *Ability to reach:* comprehensive assessment of needs was provided at home; a web site to ease access to additional support services online. *Ability to engage:* involvement in the development of individual short-term family goals.	Decrease (*p* = 0.55)		Less need for: - social support (*p* = 0.03); - help with family relationship (*p* = 0.015).
Druss, 2001 [36]	Referral of patients to primary care by mental health providers		Primary care clinic located contiguous to the mental health clinics		Development of the individualized care plan according to the needs of patients.		Decrease (8.5 % vs 18 %; *p* = 0.12)	Decrease (11.9 % vs 26.2 %; *p* = 0.04)	Fewer problems with: - access to care (*p* < 0.01); - attention to patient preferences (*p* = 0.03); - courtesy (*p* = 0.046); - coordination of services (*p* = 0.01); - continuity of care (*p* < 0.001).
Counsell, 2007 [38]	Patients referred by PCPs				Individualized care plans were developed according to the needs	*Ability to engage:* patients were actively involved in the development of care plan	No difference except for high risk patients (decrease, *p* = 0.03).	Decrease (*p* = 0.03)	
Callahan, 2006 [51]	Patients referred by PCPs				Individualized care plans were developed according to the needs	*Ability to engage:* patients were actively involved in the development of care plan	No difference (29.8 % vs 24.6 %, *p* = 0.59)		
Continuity of care via arrangements for follow-up									
Sin, 2004 [57]	Patients approached at discharge to make an appointment with their PCP			Intervention in public health care system	Continuous nature of care: from discharge to asthma control by PCP		Decrease at 3 (*p* = 0.53) and 6 months (*p* = 0.27) but no difference at 12 months (*p* = 0.63).		
DeHaven, 2012 [48]	Patients were contacted to be enrolled in the intervention program after ED admission		To have an access to the health services, patients have to reside in the target area zip code.	Free access to health care services				Decrease (*p* < 0.01)	
Institution incentives									
Feinglass, 2014 [28]	Uninsured residents of suburban DuPage County with a household income below 200 % of Federal Poverty Level were assigned.		Program was implemented in 45 sites across the county.	County hospitals, county government, and other foundations financially supported the program. Moreover, the Access DuPage program pays a small capitated fee to clinics and PCPs.		*Ability to pay:* only a small copayment was required for the prescribed medications.	Increase by 14 %.	Increase by 9 %.	- decrease of waiting time to see a doctor/nurse (*p* = NS); - increase of clinic working hours (*p* < 0.0001); - increase access trough the phone (*p* < 0.05); - increase of ease to get an appointment (*p* < 0.05); - increase of ease to get transportation (*p* < 0.05); - increase of receiving care participants thought they needed (e.g., blood tests, appointment with a doctor) (p < 0.0001); - increase of satisfaction to communicate with a doctor/nurse (due to language barrier) (p < 0.0001); - increase of satisfaction with explanations doctors/nurses give (p < 0.05); - increase of time healthcare professionals spend with patients (p < 0.05); - satisfaction with respect healthcare professionals show (p < 0.05)

## Appendix 3

**Table 5:** Characteristics of included studies (state/national intervention): Access dimension and outcome

Study ID	Dimensions of access of primary care services					Dimensions of ability of consumers	Outcomes		
	Approachability	Acceptability	Availability and Accommodation	Affordability	Appropriateness	Ability to (1) Perceive; (2) Seek; (3) Reach; (4) Pay; (5) Engage	Avoidable hospitalization	Avoidable ED admission	Unmet health care needs
Clinical multidisciplinary teams									
McDermott, 2004 [62]	Implementation of diabetes registers, recall and reminder systems	Delivery of services by the local indigenous health workers	Delivery of the diabetes health services in the remote indigenous communities.				- 32 % reduction of hospitalization for diabetes-related conditions (*p* = 0.012); - Decline of hospitalization from 25 % to 20 % over 3 years (2004).		
Continuity of care via case management									
Dorr, 2008 [49]	Patients referred by PCPs				Individualized care plans were developed according to the needs	*Ability to engage:* patients were actively involved in the development of care plan	Decrease (*p* = 0.55)	By 2 years of follow-up: increase (*p* = 0.02 for all patients, *p* = 0.37 for patients with diabetes)	
Gravelle, 2007 [60]	Patients identified based on the age and frequency of emergency use			No additional payment	Individualized care plans were developed according to the needs	*Ability to engage:* patients were actively involved in the development of care plan		No effect (*p* = 0.14)	
Landi, 2001 [63]	Patients referred by PCPs		Integration of all the community-based services and services provided by the health agency/municipality into one “single enter” center		Individualized care plans were developed according to the needs	*Ability to engage:* patients were actively involved in the development of care plan	Decrease by 18 % (p < 0.001)		
Institution incentives									
Addink, 2011 [58]									No large improvement in - satisfaction with phone access (2.96 % of increase); - ability to get appointment within 48 h (1.12 % of increase); - ability to book an appointment in advance (4.42 % of increase); - ability to see a particular PCP (1.21 % of increase); - satisfaction with opening hours (1.25 % of decrease).
Tan, 2012 [66]	The whole population is eligible	Services were developed with active partnership of ethnic communities (iwi)		Depending on the income level: very low fees (free to $15 for all ages), low fees ($16–$30), medium ($31–$39), high ($40 or above).		*Ability to engage:* representatives of local ethnic communities were actively involved in the development of care programs	4 % decrease over five years	Enrolled patients contributed to 0.2 % increase in comparison to 1.7 % increase of not enrolled (overall steady increase of 2 % per year).	
Capitation									
Davidoff, 2008 [25]				No payment (Medicaid and State Children’s Health insurance Program)		*Ability to pay:* no charge as these managed care programs are funded by the State.		Slight reduction (3.8 % points) (*p* = NS).	No effect on unmet medical care needs

## Abbreviations

EPOC: Cochrane Effective Practice and Organization of Care Review Group
IMPACT: Improving Models Promoting Access-to-Care Transformation
OECD: Organization for Economic Cooperation and Development
